# mtDNA Single-Nucleotide Variants Associated with Type 2 Diabetes

**DOI:** 10.3390/cimb45110548

**Published:** 2023-10-30

**Authors:** Enrique Garcia-Gaona, Alhelí García-Gregorio, Camila García-Jiménez, Mildred Alejandra López-Olaiz, Paola Mendoza-Ramírez, Daniel Fernandez-Guzman, Rolando Alberto Pillado-Sánchez, Axel David Soto-Pacheco, Laura Yareni-Zuñiga, María Guadalupe Sánchez-Parada, Ana Elizabeth González-Santiago, Luis Miguel Román-Pintos, Rolando Castañeda-Arellano, Luis Daniel Hernández-Ortega, Arieh Roldán Mercado-Sesma, Felipe de Jesús Orozco-Luna, Carlos Villa-Angulo, Rafael Villa-Angulo, Raúl C. Baptista-Rosas

**Affiliations:** 1 Facultad de Medicina, Benemérita Universidad Autónoma de Puebla, Puebla 72420, Mexico; enrique.garciaga@alumno.buap.mx; 2 Facultad de Enfermería Región Poza Rica-Tuxpan, Universidad Veracruzana, Veracruz 91700, Mexico; zs18005332@estudiantes.uv.mx; 3 Facultad de Ciencias Médicas y Biológicas “Dr. Ignacio Chávez”, Universidad Michoacana de San Nicolás de Hidalgo, Morelia 58000, Mexico; 1593027a@umich.mx; 4 Escuela de Nutrición, Universidad del Valle de Atemajac, León 37330, Mexico; milyolaiz@gmail.com; 5 Facultad de Ciencias Biológicas, Benemérita Universidad Autónoma de Puebla, Puebla 72420, Mexico; paola.mendozaramirez1@gmail.com; 6 Carrera de Medicina Humana, Universidad Científica del Sur, Lima 15067, Peru; dfernandezg@cientifica.edu.pe; 7 Facultad de Medicina Extensión Los Mochis, Universidad Autónoma de Sinaloa, Sinaloa 81223, Mexico; rolandops20@ms.uas.edu.mx (R.A.P.-S.); axelsoto.fm@uas.edu.mx (A.D.S.-P.); 8 Departamento de Ciencias de la Salud-Enfermedad como Proceso Individual, Centro Universitario de Tonalá, Universidad de Guadalajara, Tonalá 45425, Mexico; lauray.zuniga@academicos.udg.mx (L.Y.-Z.); luis.roman@acaemicos.udg.mx (L.M.R.-P.); arieh.mercado@academicos.udg.mx (A.R.M.-S.); 9 Departamento de Ciencias Biomédicas, Centro Universitario de Tonalá, Universidad de Guadalajara, Tonalá 45425, Mexico; maria.sparada@academicos.udg.mx (M.G.S.-P.); ana.gonzalez@academicos.udg.mx (A.E.G.-S.); rolando.castaneda@academicos.udg.mx (R.C.-A.); luis.hortega@academicos.udg.mx (L.D.H.-O.); 10 Centro de Investigación Multidisciplinaria en Salud, Universidad de Guadalajara, Tonalá 45425, Mexico; 11 Centro de Análisis de Datos y Supercómputo, Universidad de Guadalajara, Tonalá 45425, Mexico; felipe.orozco@academicos.udg.mx; 12 Laboratorio de Bioinformática y Biofotónica, Instituto de Ingeniería Universidad Autónoma de Baja California, Mexicali 21100, Mexico; villac@uabc.edu.mx (C.V.-A.); rafael.villa@uabc.edu.mx (R.V.-A.); 13 Hospital General de Occidente, Secretaría de Salud Jalisco, Zapopan 45170, Mexico

**Keywords:** mtDNA, mitogenome, type 2 diabetes, variant, polymorphism

## Abstract

Type 2 diabetes (T2D) is a chronic systemic disease with a complex etiology, characterized by insulin resistance and mitochondrial dysfunction in various cell tissues. To explore this relationship, we conducted a secondary analysis of complete mtDNA sequences from 1261 T2D patients and 1105 control individuals. Our findings revealed significant associations between certain single-nucleotide polymorphisms (SNPs) and T2D. Notably, the variants m.1438A>G (rs2001030) (controls: 32 [27.6%], T2D: 84 [72.4%]; OR: 2.46; 95%CI: 1.64–3.78; *p* < 0.001), m.14766C>T (rs193302980) (controls: 498 [36.9%], T2D: 853 [63.1%]; OR: 2.57, 95%CI: 2.18–3.04, *p* < 0.001), and m.16519T>C (rs3937033) (controls: 363 [43.4%], T2D: 474 [56.6%]; OR: 1.24, 95%CI: 1.05–1.47, *p* = 0.012) were significantly associated with the likelihood of developing diabetes. The variant m.16189T>C (rs28693675), which has been previously documented in several studies across diverse populations, showed no association with T2D in our analysis (controls: 148 [13.39] T2D: 171 [13.56%]; OR: 1.03; 95%CI: 0.815–1.31; *p* = 0.83). These results provide evidence suggesting a link between specific mtDNA polymorphisms and T2D, possibly related to association rules, topological patterns, and three-dimensional conformations associated with regions where changes occur, rather than specific point mutations in the sequence.

## 1. Introduction

Type 2 diabetes (T2D) is the most prevalent metabolic disease and a significant public health concern [1,2], leading to early-onset disability and elevated mortality due to various related complications [3]. Between 1990 and 2017, the global trend in the age-standardized rates of T2D, as measured by the number of cases per 100,000 population, showed a significant increase. Specifically, the incidence rose from 228.5 (213.7–244.3) to 279.1 (256.6–304.3), the prevalence climbed from 4576.7 (4238.6–4941.9) to 5722.1 (5238.2–6291.0), and mortality increased from 10 (9.5–10.6) to 13.2 (12.7–13.7). Additionally, disability-adjusted life years (DALYs) grew from 553.6 (435.1–696.5) to 709.6 (557.2–888.3) [4]. Although the rise in T2D is attributed to a complex interplay between environmental factors, genetic components that heighten the risk of T2D have also been pinpointed [5].

The prevalence of T2D varies widely between populations, from a small percentage among Caucasians in Europe to more than 50% among the Pima community in Arizona. Although environmental and cultural factors account for some of this observed ethnic disparity, genetic differences also play a role [5]. Notably, T2D is often diagnosed at younger ages and a lower BMI in males. Conversely, when associated with obesity, it is more prevalent among female patients [6,7].

Insulin resistance is essential for the development of T2D and is present in most individuals with carbohydrate metabolism disorders before developing clinical manifestations that can be classified, according to current criteria, as T2D. This very important metabolic problem has hereditary determinants that are not well established so far, as well as environmental determinants focused on energy storage and metabolism. Recent knowledge from genomic analyses in humans, together with in vivo and ex vivo metabolic studies in cell and animal models, has evidenced the critical importance of the role of reduced mitochondrial function as a predisposing condition for insulin resistance [8]. These studies support the hypothesis that reduced mitochondrial function, particularly in insulin-responsive cells such as skeletal muscle fibers, adipocytes, and hepatocytes, is inextricably linked to insulin resistance through effects on the insulin balance of cellular energy [9]. In the setting of the diabetic patient, basal insulin secretion is increased. However, after a certain time, pancreatic beta cells fail to compensate for this abnormality, leading to clinical hyperglycemia, with the skeletal muscle accounting for the most insulin-stimulated glucose disposal; consequently, it is the predominant insulin resistance site in T2D [10,11].

Insulin resistance diabetes’ heritability in families is well recognized, and the risk of developing it depends on genetic and environmental factors. However, heritability estimates vary between 25% and 80% in different studies; the highest estimates are seen in those studies with the most extended follow-up periods, and the exact mechanism of transmission from parents to children has not been precisely established [5,12,13]. Individuals with one diabetic parent face a 40% lifetime risk of T2D, which rises to nearly 70% if both parents have the condition. First-degree relatives with an affected parent or sibling have roughly three times the risk compared to the general population, which can increase to approximately six times if both parents are affected. Still, these statistics can fluctuate based on the specific cohort or population [5].

In recent years, mitochondrial genome polymorphisms have been the target of study in the pathogenesis and progression of different chronic metabolic diseases [14,15,16,17,18]. Mitochondria can store between 2 and 10 copies of mitochondrial DNA (mtDNA) [19], The lack of histones in DNA leads to a mutation rate that is 6 to 17 times higher than that of nuclear DNA [20]. In addition, this allows the number of mitochondria and the genetic content of the mitochondria to vary between the cell tissues of the same person. Due to this, the presence of mtDNA mutations in the tissues that participate in the regulation of metabolism can contribute to dysfunction and, therefore, to the development of metabolic diseases [21,22,23]. Thus, it has been reported that mitochondrial metabolism is involved in the processes that control insulin release from pancreatic β cells [24], and its dysfunction due to mutations in the mtDNA would favor the development of diabetes [25,26,27,28] On the other hand, it has been reported that the presence of mutations in the mtDNA of insulin target tissues (like myocytes), and consequently mitochondrial dysfunction, plays a highly debated role in the development of diabetes [29,30].

The single-nucleotide genetic variants in mtDNA could be associated with the risk of developing T2D [25], although the exact molecular mechanisms through which this would occur remain largely unknown. Likewise, the relevance of the polymorphisms detected in certain populations should be confirmed through studies with larger sample sizes and by encompassing various ethnic groups [31].

Besides carbohydrate metabolism, mitochondria use lipids for energy production, and decreased mitochondrial function is associated with ectopic adipose tissue and insulin resistance [11]. Resting adenosine triphosphate (ATP) synthesis in skeletal muscle in insulin-resistant subjects is reduced compared with insulin-sensitive individuals, suggesting a contribution of mitochondrial dysfunction to insulin resistance [32,33].

Previous studies have suggested an association between decreased muscle oxidative capacity and insulin resistance in T2D and obesity. Thus, muscle citrate synthase correlates strongly with its mitochondrial content, oxidative capacity, and reduced maximal oxygen consumption in T2D [34,35]. Furthermore, studies of key metabolic enzymes in muscle have shown an increased ratio of glycolytic capacity relative to mitochondrial oxidative capacity in patients who have T2D and a significant correlation between the ratio of glycolytic to oxidative enzyme capacity and insulin resistance [10].

Mitochondria in skeletal muscle from patients who have T2D are smaller and show an altered morphology [34,36]. The reduction in mitochondrial function in patients with type 2 diabetes is accompanied by an increase in the intracellular lipid concentration in the skeletal muscle tissue, a reduction in mitochondrial density/content, and a decrease in mitochondrial oxidative phosphorylation rates [37,38,39].

Most evidence and published studies focus on the association of mitochondrial alterations with insulin resistance, both in its number per cell and metabolism. A decrease in mitochondria number and electron transport chain activity in T2D and obese patients compared to lean volunteers has been previously documented [34,40], as well as impaired mitochondrial function in skeletal muscle obtained from obese type 2 diabetics [41]. A lack of response of muscle mitochondrial ATP production to high-dose insulin infusion has also been reported in type 2 diabetic subjects, suggesting an impaired response to insulin and reduced mitochondrial function [42,43]; a modest decrease in mitochondrial ATP synthesis rates in non-obese T2D patients and older non-diabetic individuals compared with younger non-diabetic groups under fasting conditions and after insulin stimulation [44]; and reduced basal ADP-stimulated and intrinsic mitochondrial respiratory capacity in type 2 diabetic subjects compared to control subjects matched for age and BMI [45,46,47]. However, some authors attribute the reduced mitochondrial capacity per unit mass skeletal muscle observed in T2D patients to the concept of reduced mitochondrial content and volume, oxidative enzyme levels, and mtDNA, and decreased levels of co-regulators of mitochondrial biogenesis [48]. Transcriptomic evidence of altered mitochondrial biogenesis and proteomic alterations of mitochondrial dysfunction [49,50,51] does not entirely exclude the possibility of primary defects in mitochondrial function in human skeletal muscle, and no evidence so far demonstrates a cause-and-effect relationship between insulin resistance and T2D [10].

The primary aim of this study was to conduct secondary research to ascertain the prevalence of mtDNA SNPs in individuals with and without T2D, and to investigate their potential relationship with the disease.

## 2. Materials and Methods

An analytical cross-sectional study was conducted based on complete mitochondrial genome sequences in the NCBI Nucleotide database https://www.ncbi.nlm.nih.gov/nuccore (accessed on 8 June 2021). To identify the sequences, we used booleans and keywords in the search string (015400[SLEN]:016700[SLEN]) AND (Homo[Organism] OR Homo sapiens[organism]) AND mitochondrion[FILT] AND (“Type 2 diabetes” OR “non-insulin-dependent diabetes” OR T2D). We filtered those with only complete chromosome mitochondrial sequences.

Once the sequences were identified in the database, the metadata associated with each of them were explored to validate the place of origin; the following were required as inclusion criteria: (1) sequence length of 16,569+/−10 bp, (2) *Homo sapiens* species tag, (3) type 2 diabetes diagnostic tag or defined as non-insulin-dependent, (4) tag of origin of the sequence as a control grade individual in a study published on T2D or defined as non-insulin-dependent, and (5) reference citation of the article associated with the study of the origin of the sequence, eliminating all those who did not meet any inclusion criteria. In cases where the metadata tags of each individual were not clear enough to distinguish between diabetic and control cases, the first author of each study was contacted via email, to provide the specific criteria that they used to classify each individual.

For the identification of haplogroups and polymorphisms of the selected sequences using the Genebank sequence number, the haplotype was determined and polymorphisms identified using MITOMASTER https://www.mitomap.org/foswiki/bin/view/MITOMASTER (accessed on 1 July 2021), from which a database was built with the identified polymorphisms. The criteria for the classification of the different haplogroups can be found in more detail in the Phylotree database http://www.phylotree.org/ (accessed on 1 July 2021) [52,53] In addition, for the construction of the database, the alignment of the sequences was carried out in the genomic browser UCSC Genome Browser https://genome.ucsc.edu (accessed on 10 July 2021) to be able to analyze the presence of the polymorphisms of interest in each one of the sequences manually, recording deletions, insertions, and substitutions when compared to the *rCRS* reference sequence https://www.ncbi.nlm.nih.gov/nuccore/251831106 (accessed on 20 July 2021).

**Figure 1:**
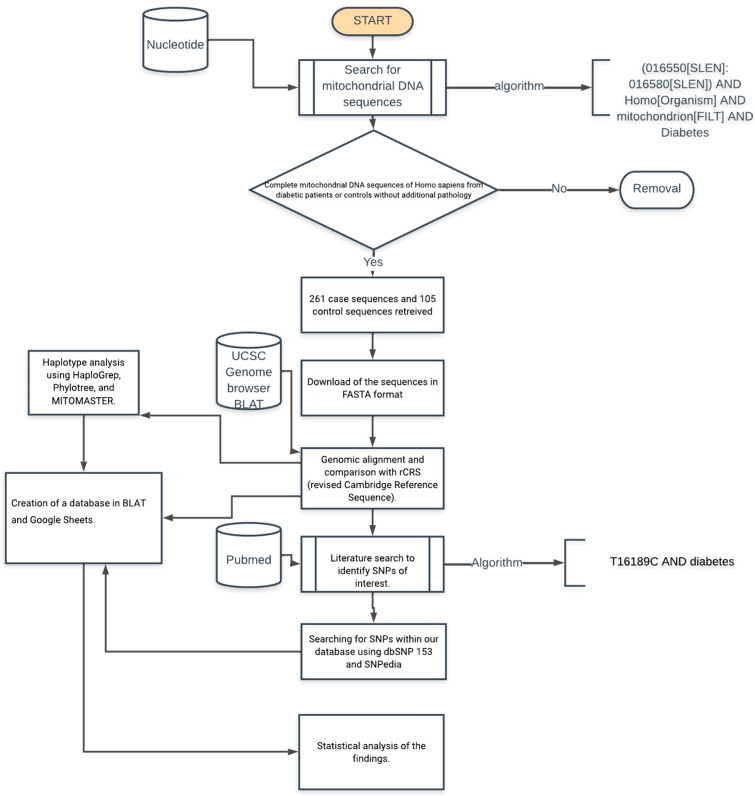
Methodological pipeline to identify and process sequences associated with type 2 diabetes and controls obtained from NCBI Nucleotide database.

We conducted a literature research to identify those SNPs that have been previously reported to be associated with T2D. This was done in NCBI PubMed, with our main research string using the variant and the keyword “Type 2 Diabetes”, reasoning that only some polymorphisms have been associated with T2D in various publications. We obtained 80 polymorphisms to be associated with T2D. The *NCBI dbsSNP* https://www.ncbi.nlm.nih.gov/snp/ (accessed on 23 July 2021) and *SNPedia* databases (https://www.snpedia.com/) were used to acquire vast information about the SNPs, such as their frequency, clinical significance, and publications previously published. The methodology used to obtain and process the sequences is summarized in Figure 1.

The databases were analyzed using R (version 4.0.3) https://cran.r-project.org/ (accessed on 1 October 2022). The absolute and relative frequencies were calculated for the descriptive analysis of the nominal qualitative variables related to the presence or absence of SNPs in a specific locus. The goodness of fit hypothesis used Pearson chi-square (*X*^2^) analysis with *p* < 0.05 and the estimated odds ratio (OR) and relative risk with their respective 95% confidence intervals (95% CI).

For the descriptive statistical analysis of counts of variants in a single sequence, we utilized central tendency statistics such as the mean, median, and mode; dispersion statistics such as the standard deviation, standard error, variance, quartiles, interquartile range (IQR), and maximum and minimum values; and finally asymmetry metrics, such as skewness and kurtosis. To explore normality, a qqplot, the Kolmogorov–Smirnov test with the Lilliefors correction, and the Pearson test were used to test the normal distribution of data. To explore variance homoscedasticity, a Levene test was performed. A comparative analysis of the median of the number of polymorphisms between diabetic and control cases was performed using the Mann–Whitney U test (Wilcoxon–Mann–Whitney test) with the criterion of *p*-value < 0.05 to confirm statistically significant differences.

A proportion test was added to compare the number of successes in independent groups and the corrected *p*-value was obtained in a Pearson’s chi-squared test with Yates continuity correction to prevent the overestimation of our data. Based on significant differences between polymorphism presence/absence in a given position in the chi-square test and post hoc test, a standardized residual test, Holm–Bonferroni adjustment, and the Tukey test were performed to control for type I error and counteract the problem of multiple comparisons. The odds ratio was calculated from the fourfold (2 × 2) contingency tables (variant presence–absence vs. T2D–control), and corrected odds ratios were estimated by logistic regression with a 95% confidence interval, without considering missing values in the calculation.

Logistic regression models were used to examine the relationships between categorical variables controlling for other variants in other positions. This allowed us to evaluate the effect of a categorical variable on the probability of a binary or categorical outcome, taking into account other predictor variables such as the associations between various polymorphisms. A multinomial logistic regression analysis was also conducted to analyze the associations between them and understand how the categories of the dependent variable, diabetes, were related to the predictor variables associated with the presence of various mitochondrial polymorphisms. Finally, from the regression model, a receiver operating characteristic (ROC) curve was constructed and the area under the curve (AUC) was estimated by calculating the sensitivity and specificity of the variants as a whole in a patient with type 2 diabetes to the estimated genetic risk score as a method to summarize individual effects.

## 3. Results

Out of 2663 mtDNA sequences found in the NCBI Nucleotide database, 2366 met the eligibility criteria. Of these, 54% (*n* = 1261) were from individuals diagnosed with T2D, while 46% (*n* = 1105) were from control individuals. The summary of the results of the descriptive statistics of the number of polymorphisms per sequence is shown in Table 1 and the frequency of different haplogroups in Supplementary Table S1. After conducting the graphical analysis and the statistical test to determine normality, it was found that the frequency distribution of variants did not follow a normal distribution (Table 1). Normality analysis with the Kolmogorov–Smirnov test with Lilliefors correction and the Pearson test demonstrated the absence of normality and the non-parametric distribution of the data (Supplementary Figure S1).

**Figure 2:**
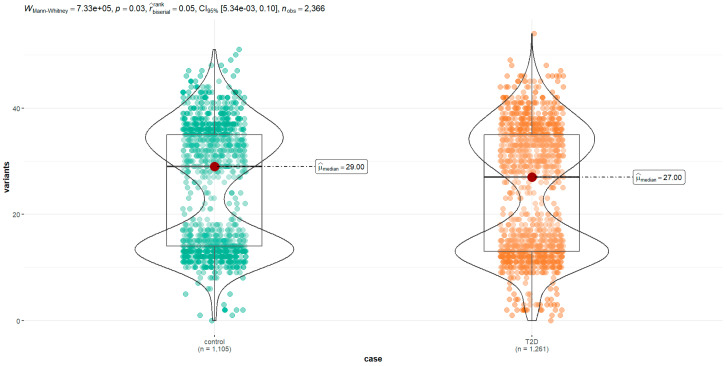
Mann–Whitney U test of frequency of mtDNA polymorphisms per sequence in 2633 cases analyzed. A significant difference was identified between the medians of the groups with a value of *p* < 0.05.

When analyzed individually by separate groups and plotted using frequency histograms of the variant counts per sequence, this bimodal distribution was maintained in diabetics and controls, so it was not an effect related to differences in the frequencies between the two groups. With the Levene test to explore the homoscedasticity of the same data, their variances were homogeneous, with an *F* statistic value of 2.21 and a value of *p* equal to 0.14. Finally, in the Mann–Whitney test, we demonstrated a significant difference when comparing the median of variants’ frequency in the diabetic group with controls (Figure 2).

The descriptive statistics analysis showed that in terms of the mean of variants in the diabetic population, most of the variation in control sequences was attributed to mainly unique polymorphisms. Only 13 mutations were related in almost 50% of the sequences studied. In contrast, there were up to 22 different polymorphisms in the control sequences. Most variations were found in unique polymorphisms distributed along the mitochondrial genome; however, the most frequent repetition was concentrated in the control region, between positions 576 and 16,024 of the mtDNA.

**Figure 3:**
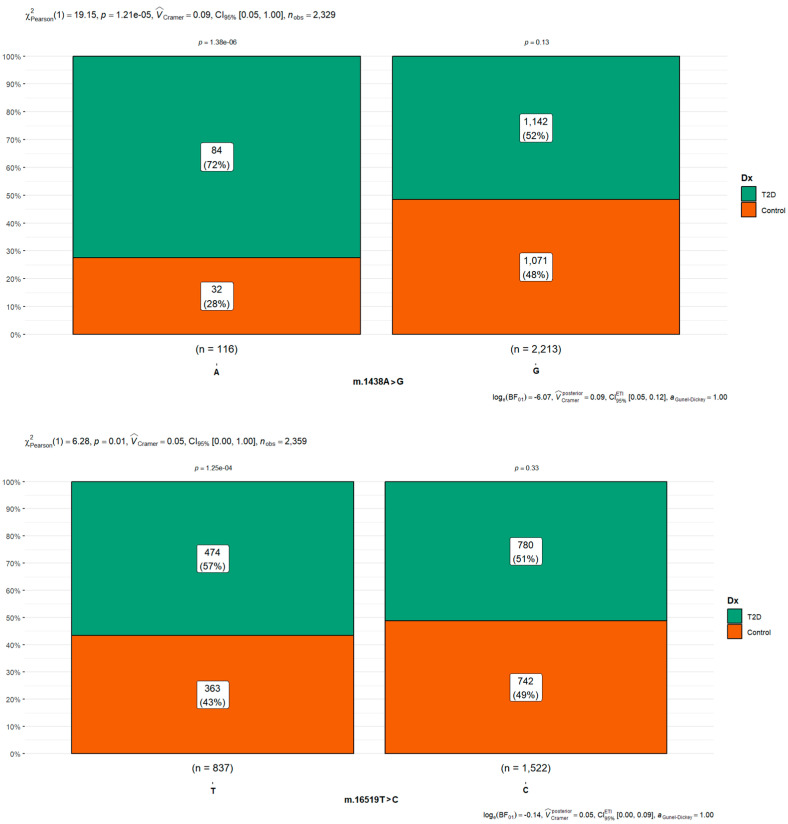
Pearson *X*^2^ with goodness of fit test of mitochondrial single-nucleotide polymorphisms m.1438A>G (rs2001030) and m.16519T>C (rs3937033) in type 2 diabetes cases and controls. This variant was the most frequent in the analysis, with a significant *p* value > 0.05.

Of the 13,907 SNPs (7087 in T2D and 6820 in controls) identified in the sequences after comparison against the *rCRS*, 64% were found to be associated with only five variants (8901 of which 4536 were in T2D cases and 4365 in T2D cases), occupying two thirds of all the polymorphisms observed in all the samples analyzed. The five most frequently shared polymorphisms were m.1438A>G (rs2001030), found in people with diabetes in 97.0% (1071/1104) of diabetic cases and 90.6% (1142/1105) of controls (Figure 3); m.750A>G (rs2853518), found in 96.1% (1211/1260) of diabetic cases and 96.2% (1071/1105) of controls; m.16519T>C (rs3937033), found in 61.8% (780/1661) of diabetic cases and 67.1% (742/1105) of controls (Figure 3); m.2706A>G (rs28541280), found in 55.5% (700/1261) of diabetic cases and 58.7% (648/1104) of controls; and m.73A>G (rs869183622), found in 56.4% (711/1261) of diabetic cases and 60.1% (663/1104) of controls. A proportion test and Pearson’s chi-square test confirmed the significant difference, as shown in Figure 3 and Supplementary Tables S2 and S3.

Of these more frequent changes found, the m.16519T>C (rs3937033) polymorphism was the only significant difference between groups with *p*-value < 0.05 in the chi-square test (Figure 3). Concerning variant m.16189T>C (rs28693675), frequently reported in the literature in multiple studies in different populations associated with T2D, it was only found in 13.6% (171/1261) of the cases with diabetes and 13.4% (148/1104) of the control cases (Figure 3). It was not found to be associated with diabetes in our analysis, with *p*-value 0.83 and odds ratio 1.03 with 95%CI 0.815–1.31. In the diabetic group, 13.7% of the sequences (187/1361) did not have SNPs, resulting in being identical to the reference sequence; each sequence had, on average, six SNPs, with a maximum of 70 variants (Figure 1). Meanwhile, in the control group, at least 15.9% (176/1105) of the sequences did not have SNPs, and there was a higher frequency of polymorphisms, with a maximum of 146 and an average of eight SNPs per sequence (Table 2). In the standardized residual test and Bonferroni adjustment of mtDNA variants associated with type 2 diabetes, statistical differences were observed with *p* value < 0.05, which are shown in Supplementary Tables S4 and S5.

Finally, we explored the risk prediction evaluation with the receiver operating characteristic (ROC) curve between mtDNA variants associated with T2D, the true positive rate as sensitivity against the false positive rate as one minus specificity, for the different possible cutoff points of the presence of variants m.1438A>G, m.14766C>T, and m.16519T>C, the most frequent variants found in our analysis. The estimated area under the curve (AUC) was 0.6340978 (Supplementary Figure S2).

## 4. Discussion

Although a well-established relationship exists between mitochondrial physiology and type 2 diabetes (T2D), mitochondrial dysfunction can impair glucose metabolism and contribute to the disease’s development [44]. Mitochondrial dysfunction is intricately linked to T2D through several pathways, which include diminished energy production, oxidative stress, the altered metabolism of fatty acids, inflammation, and aberrations in cell signaling. Mitochondria play a pivotal role in synthesizing adenosine triphosphate (ATP), which is the cellular mainstay of energy. Consequently, any dysfunction may diminish ATP production, impairing the cells’ capacity to metabolize glucose and regulate blood sugar levels effectively. Concurrently, mitochondrial dysfunction can also augment the production of reactive oxygen species, engendering oxidative stress. This stress has the potential to inflict damage on cells and tissues, notably the insulin-producing beta cells in the pancreas. Such damage could precipitate a decline in insulin secretion and foster insulin resistance, both of which are crucial characteristics of T2D [9,34,46].

Likewise, mitochondria play an essential role in the metabolism of fatty acids, an important energy source for cells. Mitochondrial dysfunction can disrupt fatty acid metabolism, contributing to ectopic lipid storage and insulin resistance. Mitochondrial dysfunction can trigger inflammatory responses in cells and contribute to the dysfunction of cell signaling pathways so that these changes may affect the function of pancreatic beta cells, as well as the response of peripheral tissues to insulin, which may contribute to the development of an intolerance to carbohydrates [54].

Although it is well known that the region of the mitochondrial chromosome with the most significant variability is the control region, where more than 90% of the changes are grouped when comparing different sequences [55,56,57,58,59,60] it is noteworthy that the identified polymorphisms are concentrated in two loci related to the synthesis of mitochondrial ribosomal subunits: four variants, m.1189T>C (rs28358571), m.1193T>C (rs111033321), m.1420T>C (rs111033356), and m.1438A>G (rs2001030), in the MT-RNR1 gene locus coding for the 12S ribosomal RNA, and three variants, m.1811A>G, m.2667T>C, and m.3027T>C, in the MT-RNR2 gene locus, coding for the 16S ribosomal RNA. This important mtDNA region is related to MDP, encoded in the mitochondrial genome and translated into the mitochondria or cell cytoplasm and released to bind to extracellular membrane receptors, with active participation in cellular metabolism as a source of regulation factors of metabolic stress.

A previous analysis of our research team reported three variants in MT-RNR1 not related to the MOTS-c coding sequence: m.1189T>C (rs28358571), m.1420T>C (rs111033356), and m.1438A>G (rs2001030). Secondly, it revealed three polymorphisms associated with MT-RNR2: m.2667T>C (rs878870626), related to humans; m.1811A>G (rs28358576) in SHPL3; and m.3027T>C (rs199838004) in SHPL6, associated with statistical differences between the T2D and control group. All these findings were previously related to cardiovascular complications in the literature and, as far as we know, have been revealed for the first time in diabetic patients [61].

The available evidence is compelling regarding the highly nuanced and bi-directional relationship between mitochondria and diabetes. On the one hand, aspects of T2D, such as insulin resistance, can lead to mitochondrial dysfunction, such as through energy overload leading to ROS excess production. Otherwise, mitochondrial dysfunction may lead to the subsequent development of T2D, as evidenced by the presence of mitochondrial SNPs associated with this metabolic disease.

Some of these pathophysiological phenomena, clearly associated with mitochondrial dysfunction, may be associated with variants in their genome and the proportion of mitochondria with these changes in essential tissues linked to the disease, such as striated muscle fibers, adipocytes, hepatocytes, and pancreatic beta cells [62,63,64,65]. In addition, the role of mitochondrial genetics in the risk of the diabetic phenotype has been established, relating it to several mtDNA changes that have been associated with the development of T2D, like heteroplasmic variants associated with an increased risk of carbohydrate intolerance, such as m.3243A>G (rs199474657), m.14577T>C (rs386829219), and m.5178A>C (rs28357984) [66,67,68,69] and homoplasmic variants that include m.1310C>T (rs111033354), m.1438GA>G (rs2001030), m.12026A>G (rs202136725), m.16189T>C (rs28693675), and 14693A>G (rs386829226) [67,70,71]. In the descriptive statistical analysis, the variants were more frequent with statistical significance in controls compared to the diabetic population. Also of note is the bimodal distribution of the number of SNPs per sequence, both in people with diabetes and in controls, the first and most important being where the mode is centralized, with 13 variants per sequence, and the second peak appearing between 30 and 35 variants by sequence. A bimodal distribution with two maximum points makes the mean and median useless, since their values will be somewhere between the two maximum points and will likely distort the distribution’s description.

This genomic bimodal architecture is found in other sites, at the same level of complexity as in the canalization phenomena [72,73] in the nuclear genome, but is also found in other levels of human DNA complexity, like in the GC proportion in the third positions of the codons, which are the ones with the greatest freedom to change without altering the encoded amino acid [74] and the genetic expression of neoplastic malignant cells [75,76,77]. In other words, the compositional characteristics of the genome constitute a genotype; in the future, this bimodal distribution of the variant frequency per sequence should be explored by analyzing a more extensive sequence set with more diverse haplogroups (Supplementary Table S1).

In our research, 80 polymorphisms of patients with T2D were analyzed. Strong prevalence was found, with significant statistical differences between diabetic and controls in three polymorphisms: m.1438A>G (rs2001030) (89.27%), m.14766C>T (rs193302980) (75%), and m.16519T>C (rs3937033).

These changes were found in different mitochondrial regions (Table 2). The m.1438A>G polymorphism (rs2001030), the most frequent change found in our analysis, is located in the MT-RNR1 gene coding for the 12S ribosomal RNA; the m.14766C>T polymorphism, also known as rs193302980, is located in the MT-CYB gene coding for the cytochrome b subunit of complex III (ubiquinol:cytochrome c oxidoreductase); m.16519T>C (rs3937033) is a variant in mitochondrial DNA in the noncoding position. In a previous comprehensive study, it was found that there was a statistically significant association between the m.16519T>C (rs3937033) variant and the prevalence of type 2 diabetes. It was observed that individuals possessing the m.16519T>C (rs3937033) variant demonstrated a 69% increase in the odds of developing T2D (OR = 1.69, CI = 1.23–2.33, *p* = 0.006) [78]. Moreover, m.10398A>G, also known as rs2853826, is found in the MT-ND3 gene coding for subunit ND3 of complex I (NADH dehydrogenase) at the first nucleotide of codon 114 (ACC) for threonine; finally, the m.16189T>C polymorphism (rs28693675) occurs in the hypervariable segment 1 (locus MT-HV1, 16024-16383) at the mitochondrial DNA replication control region. These variants have been previously investigated for different diseases and conditions, including T2D. Some studies have examined the association between these SNPs and the risk of developing the disease; however, the results have been inconsistent, and a conclusive association has not yet been established. Due to the lack of consensus on the association between our variants and type 2 diabetes, more research in different populations and more extensive studies are needed to understand their relationships better.

The m.1438 A/G polymorphism has attracted considerable attention. It stands out as the most frequently identified SNP in our analysis, observed in 97.0% (1071/1104) of diabetic cases and 90.6% (1142/1105) of controls. This variant’s significance stems from its location within the HTR2A gene, which encodes for a serotonin receptor. The sequence alteration resulting from this substitution is associated with various neuropsychiatric disorders, such as anorexia nervosa, bipolar disorder, and seasonal affective disorder. Its importance is further emphasized by its ability to modulate the activity of the HTR2A promoter [79]. Its relationship with type 2 diabetes mellitus has not been previously documented.

The m.16189T>C (rs28693675) polymorphism, located in the noncoding region of mtDNA, specifically near the segment associated with termination in the hypervariable region (HVR1), has been linked to insulin resistance and a higher prevalence of T2D, particularly in Asian, Caucasian, and Chinese populations [80,81]. While this single-nucleotide polymorphism (SNP) is thought to play a direct role in the predisposition to T2D, it is important to note that the emergence or expression of pathological mtDNA mutations may be influenced by other SNPs in the mtDNA. Examples include the mtDNA mutation m.11778G>A (rs199476112) for LHON, m.1555A>G (rs267606617) related to sensorineural deafness inherited from the mother, and m.14709T>C (rs121434453) in tRNAglu, which is recognized as a causal mutation [81].

The T-to-C transition observed in position 1989 has the potential to generate a variable-length poly-C tract. The increased number of continuous cytosines leads to a reduction in the mtDNA replication speed, resulting in a decreased count of mtDNA copies and a metabolic efficiency drawback; other factors, such as age, oxidative–antioxidative balance, and BMI, might exhibit similar effects on mtDNA copy numbers [82,83]. Although we did not observe a significant correlation of this SNP with our T2D population, the polymorphism remains noteworthy. It is the sole SNP with robust evidence supporting its role in the pathogenesis of T2D.

Finally, the m.14766 C>T (rs193302980) polymorphism is located in the MT-CYB gene coding for the cytochrome b subunit of complex III (ubiquinol:cytochrome c oxidoreductase), the second nucleotide of codon 7 (ACT) for threonine. This polymorphism has been associated with familial cancer of the breast, where its clinical significance is more likely pathogenic; on the other hand, it is also associated with Leigh syndrome, where it is significant if benign (dbSNP NCBI).

In our study, we found a prevalence rate in controls of 498 (36.9%) and in T2D of 853 (63.1%) (OR: 2.57, 95%CI: 2.18–3.04, *p* < 0.001). Our study shows the relevance of this polymorphism not only to the development of T2D but also other diseases, where it can behave as a protective or a potentially aggressive SNP.

Previous reports remark on the high prevalence in individuals belonging to haplogroup B, a highly distributed group worldwide that is recognized for its diabetogenic nature, so its study is particularly relevant in different populations also associated with other phenotypes and syndromic diseases related to carbohydrate intolerance [84,85,86]. Our analysis found higher prevalence in haplogroups R0, JT, and U. Haplogroup R0, formerly called pre-HV, is a typical Western Eurasian human mitochondrial haplogroup that evolved in Ice Age oases in South Arabia around 22,000 years ago and descended from haplogroup R, giving rise to haplogroups HV and R0a’b; haplogroup JT also has a Euroasiatic distribution and probably originated in Southwest Asia 50,300 years ago; and haplogroup U, also typical of Western Eurasia, arose from haplogroup R, likely during the early Upper Paleolithic. Recent research suggests that haplogroups R0 and J may decrease the risk of diabetes mellitus [80,87] but are related to complicated diabetic disease in other human groups [88].

These discrepancies could be attributed to differences in the populations studied, confounding factors, and methodological limitations. Mitochondrial polymorphisms, including our variants found, may have an additive effect or interact with other genetic and environmental factors to contribute to the disease risk. Given the lack of consensus on the association between SNPs and T2D, more research is needed in different populations. In the same way, other processes of analyzing nonlinear complex patterns in DNA sequences should be developed. For example, data mining techniques such as association rules can reveal hidden relationships between these variants to find the combination of mitochondrial polymorphisms that are relevant. Association rules can be used to reveal biologically relevant associations between biological information about different combinations of the presence or absence of nucleotides in known positions [89,90,91,92,93] including homoplasmic variants such as m.1310C>T (rs111033354), m.1438GA>G (rs2001030), m.12026A>G (rs202136725), m.16189T>C (rs28693675), and 14693A>G (rs386829226) [67,70,71].

## 5. Conclusions

Although the relationship between mitochondrial polymorphisms, such as SNPs, and T2D has been studied in the past, it is essential to highlight that their contribution is under constant study, and the possible associations and underlying mechanisms are still being investigated. Some studies have suggested possible associations between specific mitochondrial variants and the risk of these metabolic diseases developing. For example, SNPs in mitochondrial genes related to energy production and glucose homeostasis can affect mitochondrial function, insulin sensitivity, and glucose metabolism, influencing the susceptibility to T2D. Some examples of mitochondrial variants that have been previously reported and investigated concerning diabetes, also found in our analysis, usually are related to the mitochondrial DNA replication control region and genes that are essential to mitochondrial metabolic energy. It has been suggested that it may be associated with increased susceptibility to and a greater risk of developing diabetes. However, it is worth noting that studies on the relationship between mitochondrial variants and T2D have been inconsistent in some cases; some studies have found significant associations, while others have yet to find a clear correlation or the results have been conflicting. The complexity of diabetes as a multifactorial disease means that multiple genetic and environmental factors may contribute to its development. Furthermore, the interaction between mitochondrial and nuclear genes may also be essential in disease susceptibility.

Overall, research on the relationship between mitochondrial polymorphisms and T2D is ongoing, and further studies are needed to fully understand the underlying mechanisms and clinical relevance of these associations. It is important to note that these are only a few examples of some of the mitochondrial SNPs that have been investigated regarding T2D; the presence of these sequence changes does not necessarily imply a risk or a direct association with disease in all populations or individuals. Therefore, it is necessary to continue the investigation in this area to elucidate the genetic variations not only in mitochondrial DNA but also in an equally important region, the nuclear DNA. More research in this area would be beneficial in the refinement of personalized medicine and would contribute in public health to identifying regions and communities that have an aggregated risk for the development of this disease.

### Limitations

The limitations of this study are inherent to its retrospective nature. The anthropometric variability of each individual was not analyzed, because it was not a variable evaluated within the metadata of the literature analyzed.

The baseline characteristics of each individual, such as age, gender, BMI, and other significant characteristics such as physical activity and the use of medication, were not provided in the metadata; thus, we were not able to create a baseline characteristic table of our population.

Most of our study population belonged to haplogroup R0, which also includes haplogroups HV, H, and V, representing a total of 90.36% (*n* = 1070); specifically, the diabetic group comprised 45.84% (*n* = 578) and the control group 44.52% (*n* = 492). The population constituted by haplogroups L, M, and N was seen in a smaller proportion in our study; therefore, comparing them to each other would have resulted in a significant bias, since there would have been a pronounced gap between them. However, we expect in the future to work with a more homogeneous population; then, it will be plausible to perform a statistical comparison between the haplogroups and the presence or absence of mtDNA SNPs (Supplementary Table S1).

The presence of T2D around the world is highly heterogeneous. Currently, there have been few screening and diagnostic activities carried out in various countries to determine the significant relationship between the genetic predisposition and breed type. Regarding the data obtained in this bibliographic review, the sample analyzed was small and mostly Caucasian, which could have led to the underestimation of its prevalence and should be considered an important limitation.

## Figures and Tables

**Table 1:** Descriptive statistics of mtDNA polymorphism frequencies in 2633 sequences.

Group (n)	Min.	1st Qu.	Median	Mean	Mode	3rd Qu.	IQR	Max.	SD	Variance	Skewness	Kurtosis	*D* ^a^	*p*-Value ^a^
All (2336)	0	14.0	28.0	24.73	13.0	35.0	21	54.0	11.53	132.93	−0.01	−1.14	0.2	<2 × 10^−16^
Type 2 diabetes (1261)	0	14.0	27	24.3	13.1	35.0	22	54.0	11.7	136	0	−1.38	0.07	<2 × 10^−16^
Control (1105)	0	14.0	29	25.2	13.0	35.0	21	51.0	11.4	129	−0.02	−1.45	0.3	<2× 10^−16^

^a^ Statistic *D* and *p* value of normality test with Kolmogorov–Smirnov with Lilliefors correction. IQR Interquartile range, SD standard Deviation.

**Table 2:** Frequency of most frequent mitochondrial single nucleotide polymorphisms in type 2 diabetes and controls.

mtDNA Region	*NCBI* *dbSNP* ID	Polymorphism	Variant	All n = 2366 (%)	Controls n = 1105 (%)	T2D n = 1261 (%)	Odds Ratio	CI95%	*p* -Value
Hypervariable segment 2 (locus MT-HV2, 57-372)	-	m.73A>G	A	981 (41.5)	441 (45.0)	540 (55.0)			0.012
			G	1374 (58.1)	663 (48.3)	711 (51.7)	0.88	0.74–1.03	0.114
			Other	11 (0.46)	1 (9.09)	10 (90.9)	8.17	1.56–150.17	0.046
Gene MT-RNR1 coding for the 12S ribosomal RNA	rs28358571	m.1189T>C	T	2230 (94.3)	1035 (46.4)	1195 (53.6)			0.002
			C	120 (5.07)	68 (56.7)	52 (43.3)	0.66	0.46–0.96	0.029
			Other	16 (0.68)	2 (12.5)	14 (87.5)	6.06	1.69–38.67	0.017
	rs111033321	m.1193T>C	T	2353 (99.5)	1104 (46.9)	1249 (53.1)			0.07
			C	1 (0.04)	0 (0.00)	1 (100)	NC		
			Other	12 (0.51)	1 (8.33)	11 (91.7)	9.72	1.89–177.88	0.030
	rs111033356	m.1420T>C	T	2354 (99.5)	1104 (46.9)	1250 (53.1)			0.17
			Other	12 (0.51)	1 (8.33)	11 (91.7)	9.72	1.89–177.74	0.030
	rs2001030	m.1438A>G	A	116 (4.90)	32 (27.6)	84 (72.4)			<0.001
			G	2213 (93.5)	1071 (48.4)	1142 (51.6)	0.41	0.26–0.61	<0.001
			Other	37 (1.56)	2 (5.41)	35 (94.6)	6.67	1.88–42.53	0.012
Gene MT-RNR2 coding for the 16S ribosomal RNA	rs28358576	m.1811A>G	A	2088 (88.3)	954 (45.7)	1134 (54.3)			<0.001
			G	272 (11.5)	151 (55.5)	121 (44.5)	0.67	0.52–0.67	0.002
			Other	6 (0.25)	0 (0.00)	6 (100)	NC		
	rs878870626	m.2667T>C	T	2356 (99.6)	1104 (46.9)	1252 (53.1)			0.024
			Other	10 (0.42)	1 (10.0)	9 (90.0)	7.94	1.49–146.47	0.049
	rs199838004	m.3027T>C	T	2337 (98.8)	1099 (47.0)	1238 (53.0)			0.007
			C*	24 (1.01)	6 (25.0)	18 (75.0)	2.66	1.11–7.37	0.038
			Other	5 (0.21)	0 (0.00)	5 (100)	NC		
Gene MT-ND3 coding for subunit of complex I (NADH dehydrogenase). First nucleotide of codon 114 (ACC) for threonine.	rs2853826	m.10398A>G	A	1809 (76.5)	848 (46.9)	961 (53.1)			<0.001
			G	534 (22.6)	255 (47.8)	279 (52.2)	0.97	0.80–1.17	0.722
			Other	23 (0.97)	2 (8.70)	21 (91.3)	9.27	2.71–58.04	0.003
Gene MT-CYB coding for the cytochrome b subunit of complex III (ubiquinol:cytochrome c oxidoreductase). Third nucleotide of codon 10 (CTA) for leucine.	-	m.14766C>T	C	1351 (57.1)	498 (36.9)	853 (63.1)			0.001
			T	1011 (42.7)	607 (60.0)	404 (40.0)	0.39	0.36–0.46	0.001
			Other	4 (0.17)	0 (0.00)	4 (100)	NC		
hypervariable segment 1 (locus MT-HV1, 16024-16383)	rs147029798	m.16126T>C	T	1922 (81.2)	871 (45.3)	1051 (54.7)			0.001
			C	439 (18.6)	234 (53.3)	205 (46.7)	0.73	0.59–0.89	0.003
			Other	5 (0.21)	0 (0.00)	5 (100)	NC		
Control región Noncoding position	rs3937033	m.16519T>C	T	837 (35.4)	363 (43.4)	474 (56.6)			0.001
			C	1522 (64.3)	742 (48.8)	780 (51.2)	0.81	0.68–0.95	0.012
			Other	7 (0.30)	0 (0.00)	7 (100)	NC		

Statistically significant difference with *Chi* square goodness of fit test *p* < 0.05.

## Data Availability

The identification for access to the sequences used for this work is fully referred to in Supplementary Table S7 and is available from the NCBI Nucleotide database (https://www.ncbi.nlm.nih.gov/nuccore (accessed on 1 June 2019)).

## Supplementary Materials

The following supporting information can be downloaded at: https://www.mdpi.com/article/10.3390/cimb45110548/s1, Table S1: Frequency distribution of the different haplogroups in diabetic and control cases; Table S2: Proportion test of mtDNA variants associated with type 2 diabetes; Table S3: Pearson’s chi-squared test with Yates’ continuity correction of mtDNA variants associated with type 2 diabetes. Significant differences were identified between variants m.1438A>G and m.14766C>T as m.1438A>G and m.16519T>C. In the table, statistics are shown as *X*^2^ (*p*-value); Table S4: Standardized residual test and Bonferroni adjustment of mtDNA variants associated with type 2 diabetes; Table S5: Standardized residual test and Bonferroni adjustment between mtDNA variants associated with type 2 diabetes; Table S6: List of participants in the project during the Pacific Scientific and Technological Research Summer Program 2019–2022; Figure S1: Qqplot to explore normality in number of variants per sequence in 2366 mtDNA chromosomes obtained of type diabetes cases and controls. Kolgomorov Smirnov with Lilliefors correction obtain a D statistic equal to 0.17113 with a *p*-value < 2.2 × 10^−16^, demonstrating that the data have a non-parametric distribution outside normality. Since the data are discrete, a Pearson normality test was also performed corroborating the maximum result; Figure S2: Generalized linear model and risk prediction evaluation with receiver operating characteristic (ROC) curve between mtDNA variants associated to type 2 diabetes. True positive rate (sensitivity) against the false positive rate (1-specificity) for the different possible cut points of presence of variants m.73A>G, m.1189T>C, m.1193T>C, m.1420T>C, m.1438A>G, m.1811A>G, m.2667T>C, m.3027T>C, m.10398A>G, m.14766C>T, m.16126T>C and m.16519T>C. The estimated area under the curve (AUC) was 0.6911773.
