# Supplementary material for: mtDNA Single-Nucleotide Variants Associated with Type 2 Diabetes
Source: Curr Issues Mol Biol. 2023 Oct 30;45(11):8716–32. doi: 10.3390/cimb45110548 (PMC10670651; doi:10.3390/cimb45110548)
Supplement: Supplementary file 1 [file cimb-45-00548-s001.zip › Tables S1-S6 and FIgures S1 and S2-CIMB_2023_mtDNA_T2D_.pdf]

current issues in molecular biology

MDPI

Supplementary Materials

Table S1. Frequency distribution of the different haplogroups in diabetic and control cases.

Macrohaplogroup	ALL N=2366 (100%)	Controls N=1105 (46.7%)	T2D N=1261 (53.3%)
**Haplogroups L**			
L4	1 (0.08)	0 (0.00)	1 (0.08)
L3	2 (0.16)	0 (0.00)	2 (0.16)
**Haplogroups M**			
M (includes Q)	10 (0.86)	6 (0.54)	4 (0.32)
M7	48 (4.02)	19 (1.72)	29 (2.30)
M8 (includes C, Z)	9 (0.71)	0 (0.00)	9 (0.71)
M9 (includes E)	22 (1.80)	5 (0.45)	17 (1.35)
G	10 (0.84)	4 (0.36)	6 (0.48)
D	64 (5.32)	22 (1.99)	42 (3.33)
**Haplogroups N**			
N1 (includes I)	65 (5.52)	33 (2.99)	32 (2.54)
N2 (includes W)	33 (2.82)	18 (1.63)	15 (1.19)
N9 (includes Y)	16 (1.36)	8 (0.72)	8 (0.63)
A	13 (1.06)	3 (0.27)	10 (0.79)
X	31 (2.57)	10 (0.90)	21 (1.67)
**Haplogroups R**			
R (includes P)	7 (0.57)	1 (0.09)	6 (0.48)
R0 (includes HV, H, V)	1070 (90.36)	492 (44.52)	578 (45.84)
JT (includes J, T)	411 (34.96)	211 (19.10)	200 (15.86)
R9 (includes F)	43 (3.62)	19 (1.72)	24 (1.90)
B	49 (4.13)	22 (1.99)	27 (2.14)
U (includes K)	462 (39.23)	232 (21.00)	230 (18.24)

Table S2. Proportion test of mtDNA variants associated to type 2 diabetes.

Variant	X^2	95% IC	*p*-Value
m.1438A>G	53.326	0.5565098 0.5977890	2.826e-13*
m.14766C>T	53.326	0.5565098 0.5977890	2.826e-13*
m.16519T>C	180.18	0.3383346-0.3784139	<2.2e-16*

* Statistical difference with p value < 0.05; df = 1.

Table S3. Pearson's Chi-squared test with Yates' continuity correction of mtDNA variants associated to type 2 diabetes. Significant differences were identified between variants m.1438A>G and m.14766C>T as m.1438A>G and m.16519T>C. In the table are showed statistic as X^2(*p-value*).

Variants	m.1438A>G	m.14766C>T	m.16519T>C
m.1438A>G	-		
m.14766C>T	76.435 (<2.2e-16) *	-	
m.16519T>C	81.9 (<2.2e-16) *	1.8746 (0.171)	-

* Statistical difference with p value < 0.05; df = 1.

Table S4. Standardized residual test and Bonferroni adjustment of mtDNA variants associated to type 2 diabetes. * Statistical difference with *p* value < 0.05.

m.1438A>G

Variant	Type 2 diabetes	Control
Present	-3.1790157*	0.7386723
Absent	3.0827652	-0.7163076*

m.14776C>T

Variant	Type 2 diabetes	Control
Present	-5.310265*	6.205626
Absent	5.149487	-6.017740*

m.16519T>C

Variant	Type 2 diabetes	Control
Present	-1.552454*	1.159618
Absent	1.505451	-1.124508*

Table S5. Standardized residual test and Bonferroni adjustment between mtDNA. variants associated to type 2 diabetes. *Statistical difference with *p* value < 0.05.

m.1438A>G vs. m.14776C>T

Loci		14776	
		**C**	**T**
**1438**	**A**	5.598096	-6.541988*
	**G**	-1.300767*	1.520089

X^2= 76.435, df = 1, *p-value* = <2.2e-16.

m.1438A>G vs. m.16519T>C

Loci		16519	
		**T**	**C**
**1438**	**A**	7.139048	-5.332567*
	**G**	-1.658821*	1.239069

X^2= 76.453, df = 1, *p-value* = <2.2e-16.

m.14776C>T vs. m.16519T>C

Loci		16519	
		**T**	**C**
**14776**	**C**	0.7363876	-0.5500504*

**T**	-0.8605495*	0.6427941

X^2= 1.8746, df = 1, *p-value* = 0.171.

Table S6. List of pre graduate students who collaborated in different stages of the process and analysis carried out in this project as part of the activities during the Pacific Scientific and Technological Research Summer Program 2019-2022 https://programadelfin.org.mx, in the research lines *Automation for Detection of Heteroplasmy in Mitochondrial Sequences, Mitochondrial Genomics associated with Chronic Degenerative Diseases* and *Applications of Data Science in Health*.

Benemérita Universidad Autónoma de Puebla

Baneco Rodas Ixchel Xiadani

Bernabé Gaspar Luis Eduardo

Blanco Aguilar Maria Esther

Delgado Gaytán Alexandra Viridiana

del Valle García Tania

Diaz De Lucio Yrving Daniel

Escobedo Valdez Gloria

Gómez Rodríguez Amairani

Jaimez Alvarado Samuel

Ortiz Virgen Edwin Gerardo

Quebrado Benitez Ángel Sebastian

Reyes Ramírez Lucianny Guadalupe

Romero Balboa Osorio Adriana

Váquez Páez María Fernanda

Yarce Cabrera Luis Miguel

Instituto Politécnico Nacional (Ciudad de México)

Flores Reyes Ana Karen

González Sánchez Alhelí Rocío

Juarez HernandezMisael Alejandro

Vélez Solís Carlos Alberto

Instituto Tecnológico de Los Mochis (Sinaloa)

Lugo Martinez Perla Yareli

Ruelas Ortiz Daniela

Velázquez Mercado Mariesther del Rosario

Universidad Autónoma de Baja California

Sara María Zárate Soto

Universidad Autónoma de Chiapas

Ballardo Vega Nancy Yrasema

Méndez Hernández Dulce Paola

Universidad Autónoma de Occidente (Sinaloa)

Astorga Leal Fátima
Báez Cervantes Sanely
Castro Leal Lilia Judith
Luna Valdez María Angélica
Pacheco Álvarez Astrid
Santana Quintero Luis Fernando
Zamudio Ahumada Reyna Consuelo

Universidad Autónoma de Tamaulipas
Amaro Diaz Samantha Guadalupe
Hernández Luna Jesús Alberto

Universidad Autónoma del Estado de México
del Moral Bastida Jesús Armando
Vargas Contreras María José
Alvarez Nava Mario
Valdez Romero Juan Jesus
Rodriguez Piña Miriam Deyanira

Universidad de Guadalajara
Acosta Romero Ernesto
Alvarez Martínez José de Jesús
Ascencio Reyes Juan Manuel
Barrera LujánXimena Paola
Campos Murillo Alejandra
Domínguez Sandoval María Fátima
Garcia Navarro Cesar Oswaldo
Gaspar Zuno Jesús Salvador
Gómez Castellanos Víctor Eduardo
Guizar López Rafael
Gutiérrez Luévanos Josué
Guzmán Vázquez Juan José
Jiménez Zermeño Lizbeth Deyanira
Mariscal Quirarte Karla Alejandra
Martínez Merlo David
Medina España Nayeli Tatiana
Mendoza Prado Jessica Verónica
Meraza Abarca Carlos Daniel
Morales Hernández Andrea Anahí
Muñoz Navarro Krystal Sofía
Pedroza Mata María Guadalupe
Sánchez Gutiérrez María Fernanda

Sierra Picos María Fernanda
Vargas Oceguera Erick Alejandro
Velasco Pérez Nidia Guadalupe
Xicotencatl Soto Elizabeth Patricia

Universidad Autónoma de Guerrero
Jesus Alberto Meza Hernandez

Universidad Autónoma de Sinaloa
Ballardo Vega Nancy Yrasema
Lao García Angelina
Luque Camacho Carmen Alejandra
Moraila García Beatriz Guadalupe
Rábago Monzón Juan Eduardo
Romero Iturrios Jaciel Eduardo
Soto Gómez Dzidzielia Dejanira
Torres Melchor Erika Liliana

Universidad de Monterrey
Saavedra Hernández Annel
Eugenia Urbina Arias Natalia

Universidad de Sonora
Carranza Cruz Vania Sofia

Universidad Popular Autónoma del Estado de Puebla
Contreras Cerón Luis Manuel

Universidad Veracruzana
Velazquez Melchor Mariana

Universidad Vizcaya de las Américas (Mérida, Yucatán)
Sierra Tuz_Shelby Christel

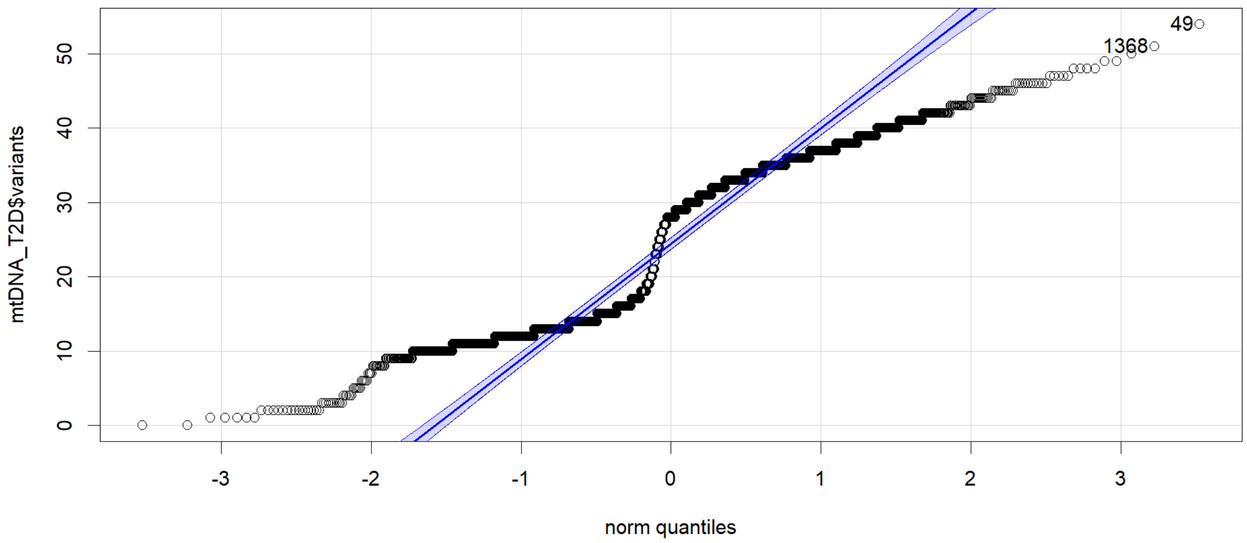

Figure S1. Qqplot to explore normality in number of variants per sequence in 2366. mtDNA chromosomes obtained of type diabetes cases and controls. Kolgomorov Smirnov with Lilliefors correction obtain a *D* statistic equal to 0.17113 with a *p-value* < 2.2e-16, demonstrating that the data have a non-parametric distribution outside normality. Since the data are discrete, a Pearson normality test was also performed corroborating the maximum result.

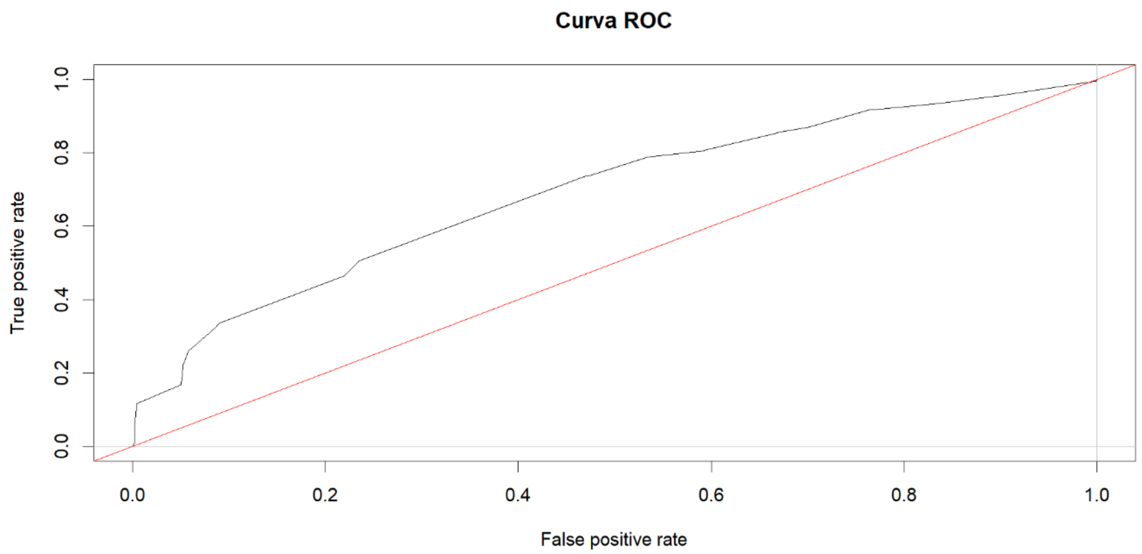

Call: glm(formula = Dx ~ m.73A>G + m.1189T>C + m.1193T>C + m.1420T>C + m.1438A.>G + m.1811A>G + m.2667T>C + m.3027T>C + m.10398A>G + m.14766C>T + m.16126T>C + m.16519T>C, family = binomial, data = data_base)

Deviance Residuals: Min -2.2363 , 1Q -1.1391, Median 0.5615, 3Q 1.1718, Max 2.1528.

Coefficients: (2 not defined because of singularities)

Estimate Std. Error z value Pr(>|z|)

(Intercept) 0.96212 0.21050 4.571 4.86e-06 ***

m.73A.G 1.43941 0.16153 8.911 < 2e-16 ***

m.1189T.C -0.45868 0.30003 -1.529 0.126323

m.1193T.C	10.92498	324.74374	0.034	0.973163	
m.1420T.C	NA	NA	NA	NA	
m.1438A.G2	-0.76045	0.22262	-3.416	0.000636	***
m.1811A.G	-0.25097	0.20613	-1.218	0.223395	
m.2667T.C NA	NA	NA	NA		
m.3027T.C	0.46311	0.52232	0.887	0.375265	
m.10398A.G	0.49912	0.13907	3.589	0.000332	***
m.14766C.T2	-2.04259	0.15259	-13.386	< 2e-16	***
m.16126T.C	-0.37249	0.13756	-2.708	0.006773	**
m.16519T.C2	-0.18837	0.09758	-1.931	0.053544	

Signif. codes: 0 *** 0.001** 0.01* 0.05

(Dispersion parameter for binomial family taken to be 1)

Null deviance: 3110.1 on 2244 degrees of freedom.

Residual deviance: 2838.9 on 2234 degrees of freedom, AIC: 2860.9

Figure S2. Generalized linear model and risk prediction evaluation with receiver operating characteristic (ROC) curve between mtDNA variants associated to type 2 diabetes. True positive rate (sensitivity) against the false positive rate (1-specificity) for the different possible cut points of presence of variants m.73A>G, m.1189T>C, m.1193T>C, m.1420T>C, m.1438A>G, m.1811A>G, m.2667T>C, m.3027T>C, m.10398A>G, m.14766C>T, m.16126T>C and m.16519T>C. The estimated area under the curve (AUC) was 0.6911773.
